# Cystic Lung and Hepatic Lesions in Hydatid Disease

**DOI:** 10.1590/0037-8682-0442-2025

**Published:** 2026-02-06

**Authors:** Danilo de Oliveira Santana Ramos, André Vaz, Vinícius Cardoso Serra

**Affiliations:** 1 Universidade de São Paulo, Faculdade de Medicina, Hospital das Clínicas, Departamento de Radiologia, São Paulo, SP, Brasil.

**FIGURE 1:**
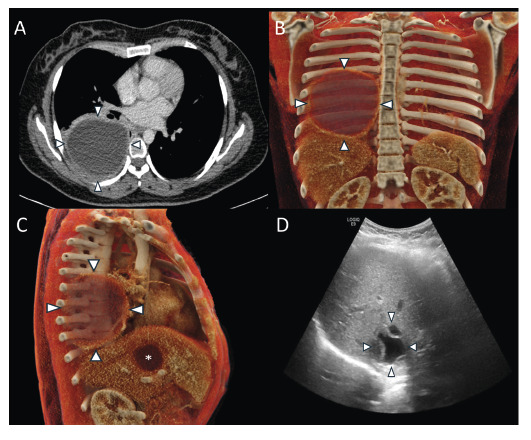
**(A)** Axial contrast-enhanced chest CT shows a large, well-defined cystic lesion in the right lower lobe of the lung (white arrowheads). **(B,C)** Cinematic volume-rendered reconstructions redemonstrate the lung (white arrowheads) and liver (asterisk) cystic lesions. **(D)** Abdominal ultrasound showing a well-defined cystic lesion in the right hepatic lobe containing a detached, irregular laminated membrane floating within its contents, consistent with the “water lily sign.”

A 24-year-old woman from Bolivia, residing in Brazil for the past 10 years, presented with intermittent back pain and occasional nonproductive cough. She reported no fever, night sweats, or hemoptysis but described an unintentional weight loss of 3 kg over the preceding months. Multimodal imaging (Figure 1) revealed large well-defined cystic lesions in the right lung and liver. Based on the patient’s clinical and epidemiologic backgrounds and radiologic findings, hydatid disease was suspected and subsequently confirmed by the detection of serum immunoglobulin G antibodies against *Echinococcus* species. The pulmonary cyst was removed via elective thoracotomy, and histopathological analysis confirmed the diagnosis. Hepatic cyst was treated by percutaneous drainage. The patient recovered uneventfully and remained asymptomatic at 1-year follow-up.

Hydatid disease, also known as echinococcosis, is a parasitic infection caused by the Echinococcus granulosus tapeworm, which is endemic in parts of South America, Africa, and Asia^1^ ^,^ ^2^. Although the disease can be multisystemic, the liver and lungs are the most frequently involved organs^2^ ^,^ ^3^. Definitive treatment generally involves surgical resection because of the low complication and recurrence rates. Antiparasitic medications such as albendazole are often used as supplementary therapy, and percutaneous methods may be suitable for selecting hepatic cases. Imaging is crucial for early diagnosis, as it helps prevent complications such as rupture, secondary infection, or spread, and assists in timely organ-preserving interventions, especially in patients from endemic areas, where hydatid disease should remain on the list of differential diagnoses.
